# Two Cases of Feather Dystrophy in Free-Living Griffon Vultures (*Gyps fulvus fulvus*) Associated with Viral-like Inclusion Bodies

**DOI:** 10.3390/ani15152190

**Published:** 2025-07-25

**Authors:** Stefano Pesaro, Donatella Volpatti, Alice Baggio, Ranieri Verin, Fulvio Genero, Luca Sicuro, Livio Galosi, Lucia Biagini, Isabella Perlin, Patrizia Robino, Barbara Colitti, Daniele Avanzato, Giacomo Rossi

**Affiliations:** 1 Department of Agricultural, Food, Environmental and Animal Sciences, University of Udine, 33100 Udine, Italy; stefano.pesaro@uniud.it (S.P.); donatella.volpatti@uniud.it (D.V.); alice.baggio@uniud.it (A.B.); isabella.perlin@uniud.it (I.P.); 2 Department of Veterinary Sciences, University of Pisa, Viale Delle Piagge 2, 56124 Pisa, Italy; ranieri.verin@unipi.it; 3 Cornino Lake Regional Natural Reserve, 33030 Forgaria nel Friuli, Italy; fulvio.genero@gmail.com (F.G.); luca@pavees.it (L.S.); 4 School of Biosciences and Veterinary Medicine, University of Camerino, 62024 Matelica, Italy; livio.galosi@unicam.it (L.G.); giacomo.rossi@unicam.it (G.R.); 5 Department of Veterinary Sciences, University of Turin, 10095 Turin, Italy; patrizia.robino@unito.it (P.R.); barbara.colitti@unito.it (B.C.); daniele.avanzato@unito.it (D.A.)

**Keywords:** griffon vulture, birds of prey, feathers abnormalities, avian polyomavirus, inclusion bodies

## Abstract

The griffon vulture (*Gyps fulvus fulvus*) is a scavenger species that plays a vital ecological role in carrion removal. Successful survival and reproduction in captive and wildlife conditions require optimal physical status and plumage integrity. Nutritional and environmental factors, systemic diseases, and various etiological agents can influence feather alterations. Although frequently documented in captive psittacine species, feather abnormalities are extremely rare in wild birds. Since 2020, two free-living griffon vultures in northeastern Italy have been found in poor physical condition, unable to fly due to partial feather loss and malformation of remiges and rectrices. Histopathologic examination of follicles and peri-follicular tissue revealed atrophy, keratin replacement, vasculitis, and calamus dystrophy with lymphohistiocytic perivasculitis. Immunohistochemical and ultrastructural analysis identified the presence of virus-like particles in epithelial and inflammatory cells. Although virome analysis did not confirm the presence of this virus in pooled affected samples, this study provides the first report of an emerging plumage disorder in free-ranging griffon vultures, which requires further characterization.

## Simple Summary

Griffon vultures are important scavengers that help maintain ecosystem balance. Two wild griffon vultures in northeastern Italy were found unable to fly due to unusual feather loss and deformities; the first was in 2020 and the second was in 2023. These abnormalities, found only in their flight feathers, were examined using histological, immunohistochemical and ultrastructural analysis and revealed signs of inflammation and viral-like particles in their feather cells. While no confirmed virus was identified through genome sequencing, the evidence points to a possible polyomavirus infection, showing a similar pathology to pet birds. This is the first report of such a condition in free-ranging vultures, raising concern for conservation efforts, as affected birds may not survive or be released into the wild. More research is needed to understand the cause and impact of this disease.

## 1. Introduction

Feathers are a distinctive and unique feature that differentiate birds from other vertebrate classes [1]. Feathers perform a variety of functions, including flight capacity, insulation, waterproofing, and camouflage, and they facilitate communication between conspecifics. Feather abnormalities can result from a wide variety of potential causes, including infectious agents, toxins, nutritional deficiencies, neoplastic diseases, immune-mediated disorders, metabolic and endocrine imbalances, behavioral issues, trauma, and management-related factors [2] as well as hormonal imbalances [3]. While generalized feather abnormalities are commonly reported in pet birds, particularly psittacine species [4,5], they are exceedingly rare in wild populations [6,7,8], especially among birds of prey. To date, the only reports of dystrophic feather development in free-living raptors in Africa and Europe include white-tailed sea eagles (*Haliaeetus albicilla*), northern goshawks *(Accipiter gentilis*), lizard buzzards (*Kaupifalco monogrammicus*), and common buzzards (*Buteo buteo*) exhibiting “Pinching Off Syndrome” (POS), a condition with unclear etiology [9]. In North America, similar alterations were observed in 2009 [10] in prairie falcons (*Falco mexicanus*), northern harriers (*Circus cyaneus*), and rough-legged hawks (*Buteo lagopus*) and were attributed to naturally acquired West Nile Virus infection. In Australia, similar abnormalities were reported during a PBFD (Psittacine Beak and Feather Disease) infection in the powerful owl (*Ninox strenua*) [11]. In the context of reintroduction and conservation programs for endangered or highly threatened avian species, plumage disorders represent a critical concern, given their potential to compromise an individual’s survival and adaptability in the wild [12,13].

Vultures are large birds of prey that occupy the pinnacle of the food chain. Vultures represent a grouping of 23 distinct species and include birds with scavenging activity [14]. The griffon vulture (*Gyps fulvus*), along with other vultures, plays an essential role in the conservation of biodiversity. It acts as a scavenger in different regions worldwide, contributing to the control of dead animal populations, the prevention of the spread of disease, and the maintenance of a healthy and balanced ecosystem. *G. fulvus*, otherwise referred to as the Eurasian griffon, is present in several European countries, with the vast majority of its population, approximately 95%, located in Spain [15]. The remaining 5% are distributed throughout the Balkan region, including Croatia, Greece, North Macedonia, Bulgaria, Serbia, France, and Italy. Its Italian breeding populations are located in Sardinia, Abruzzo, Calabria, Sicily, and Friuli-Venezia Giulia regions. The species *G. fulvus,* along with numerous others, is subjected to conservation strategies [16]. A plethora of factors, including poisoning, collision with energy infrastructure, disturbance, and habitat alteration, have led to a marked decline in the status of the griffon’s population [17]. Consequently, the ongoing monitoring of the health status of wild populations, in conjunction with the implementation of effective reintroduction strategies for endangered species, particularly those facing severe threats and a high risk of local extinction, is imperative for the conservation of biodiversity.

The objective of this study is to provide the first description of a condition characterized by feather abnormalities observed in two adult, free-living griffon vultures (*Gyps fulvus*) captured in the feeding area of Friuli-Venezia Giulia, northeastern Italy, for banding/ringing within a local population monitoring project. A detailed description of the gross and microscopical changes observed is here provided, linking the pathological scenario to a likely viral etiology. 

## 2. Materials and Methods

In 2020 and 2023, two adult free-living griffon vultures (*Gyps fulvus fulvus*) were found and captured in a feeding area of Friuli Venezia Giulia, northeastern Italy, unable to fly due to feather abnormalities. The area is frequented by other European vulture species, including the cinereous vulture (*Aegypius monachus*), the Egyptian vulture (*Neophron percnopterus*), and other necrophagous birds. Following immobilization, both animals were held in captivity and subjected to comprehensive clinical evaluations. 

### 2.1. Sampling

Wing and tail feathers, including their follicles, were surgically sampled from both vultures under inhalation anesthesia (isoflurane) for bacteriological, microscopic, immunohistochemical, ultrastructural, and molecular examinations. Blood samples were also collected. 

### 2.2. Serological Examination

In order to exclude concomitant infections, the sera of the two subjects affected by the condition were tested using specific ELISA kits to rule out the presence of antibodies against New Castel disease (ABX364913, Abbexa, Cambridge, UK), avian influenza virus (ABX092028, Abbexa, Cambridge, UK), and Flavivirus (ID Screen^®^ Flavivirus Competition, Innovative Diagnostic, Grabels, France). All tests were performed as indicated by the manufacturer. The absorbance reading was performed using the reader Infinite^®^ M Nano (Tecan, Männedorf, Switzerland).

### 2.3. Microscopical and Immunohistochemical Examination

Feathers and their follicles were excised, fixed in 10% buffered formalin, and embedded in paraffin. Tissue sections, 3 µm thick, were stained with hematoxylin and eosin for histological evaluation, and additional sections were placed on positively charged glass slides (Superfrost Plus, Fisher, Pittsburgh, PA, USA) for immunohistochemical examination, according to standard methods [18,19]. Based on the morphology of intranuclear inclusion bodies, the tissues were tested for the presence of avian polyomavirus (APV) or laryngotracheitis alpha1-*herpesvirus* (ILTV) antigens. To this end, sections were routinely deparaffinized and immersed in distilled water containing 15% hydrogen peroxide for 30 min at room temperature to block endogenous peroxidase activity. Slides were then rinsed three times for 2 min each in phosphate-buffered saline (PBS). To reduce nonspecific background staining, the sections were subsequently incubated with normal serum, obtained from the species corresponding to the secondary antibody, and diluted in a solution of 1% bovine serum albumin, 1% polyvinylpyrrolidone, and 1% tris-buffered saline (BSA-PVP-TBS) for 1 h at room temperature (RT). The sections were then incubated with primary mouse anti-VP-1 antibody, diluted 1:500 in BSA, PVP, TBS 0.1%, and rabbit anti-ILTV glycoprotein E polyclonal antibody (Cat. No. BS-1674R, Bioss, Woburn, MA, USA) diluted 1:400 in the same buffer [20]. Following overnight incubation at 4 °C in a humidified chamber, the slides were rinsed in PBS and subsequently incubated with a biotinylated anti-mouse or anti-rabbit secondary antibody for 45 min at room temperature. After three rinses in PBS, sections were incubated with enzyme conjugate (ABC-peroxidase complex, Vector, Burlingame, UK) for 45 min at room temperature, followed by incubation with 3,3′-diaminobenzidine solution containing H_2_0_2_ as substrate-chromogen for 5 min. The slides were rinsed in distilled water, rapidly counterstained with Harris hematoxylin, and examined microscopically. Negative controls consisted of normal avian tissues, while positive controls included tissues from passerine and psittacine birds infected with APV, as well as chickens or birds of prey infected with ILTV-α1-*herpesvirus*. 

### 2.4. Ultrastructural Examination

In addition to histological and immunohistochemical analyses, portions of feathers and follicles were processed for ultrastructural examination. For ultrastructural examination, the samples were immediately fixed in phosphate buffered 0.1 M 2%–glutaraldehyde pH 7.4, postfixed in phosphate-buffered 1%-OsO4, and after dehydration, embedded in Epon/Araldite (Polyscience Inc., Warrington, PA, USA). Semithin sections were stained with methylene blue and Azur II. Grids were then fixed in 1% glutaraldehyde and stained with uranyl acetate and lead citrate and examined with a JEOL 1200-EX transmission electron microscope (JEOL). Control procedures included the use of known *polyomavirus* or α1-*herpesvirus*-infected tissue. Moreover, as a control for the normal structure of the feather, healthy feathers were collected under the same conditions from the animals under study. 

### 2.5. Viral Genome Extraction and NGS Sequencing

In order to increase the likelihood of viral discovery, the following samples belonging to the two affected animals were pooled in equal weight for tissue: a pool of feathers, a pool of calamus, and a pool of blood. While tissues from specimen no. 1 were already fixed in formalin and embedded in paraffin (FFPE), tissues from the second were collected and immediately frozen at −80 °C until extraction. All the samples were subjected to virome discovery protocol through NGS sequencing. Briefly, after a homogenization step with a tissue rupture device (Qiagen, Hilden, Germany), viral DNA and RNA were extracted from the three pooled feather, calamus, and blood samples using the Qiagen Viral RNA mini kit (Qiagen, Hilden, Germany) and were fluorometrically quantified with a Qbit 3.0 instrument (Life technologies).

Viral RNA was reverse transcribed into cDNA using the Maxima H-Minus dsDNA synthesis kit (Thermofisher Scientific, Waltham, MA, USA) following the manufacturer’s instructions. Library preparation was obtained from viral DNA and cDNA using Illumina Library Prep kit (Illumina, San Diego, CA, USA) and sequenced with the Illumina Miseq platform and v3-600 cycles chemistry.

Sequencing reads were trimmed and checked for quality using Trimmomatic (version 0.39). Paired end clean reads were aligned to the *Gyps fulvus* reference genome (NCBI database nr ASM2572771v1) with Bowtie2 (v2.3.5.1) [21] and Samtools (v.1.5) [22], using the default parameters, to filter and remove host genome contamination. The remaining reads were de novo assembled using Spades (v3.14.0) [23], and contigs were aligned to the NCBI non-redundant protein database (nr20200210 version) and DIAMOND_viral_database using Diamond BLASTx (v2.0.13) [24]. The taxonomic distribution of the classified reads was visualized using Krona (v.2.7.1) [25].

## 3. Results

### 3.1. Macroscopic Examination

**Figure 1:**
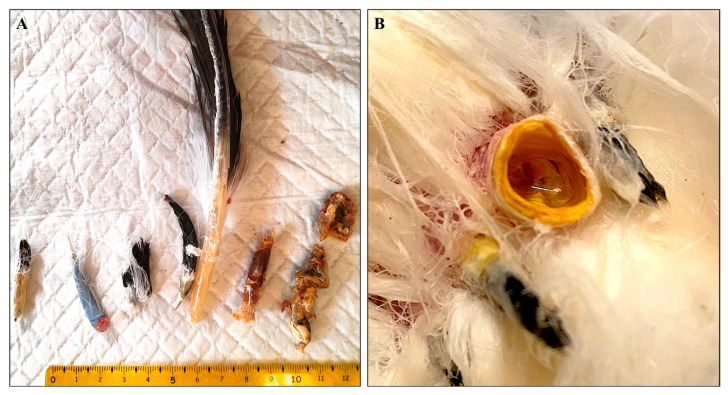
Gross aspect of altered tail feathers. (**A**) Injury patterns of differing severity, ranging from mild manifestations with alterations affecting the barbs of the vane and a markedly curved rachis, to an interruption of growth in the early stages of development due to necrosis of vascular structures, up to the formation of encapsulated amorphous material inducing follicular structural deformity. (**B**) Follicular dilation observed in the tail feathers.

Regarding the two adult griffon vultures captured and sampled, both presented poor body condition, with mild atrophy of the pectoral muscles and absent or poorly bilateral developed primary and secondary remiges. Concurrently, the rectrices displayed either absence or abnormal development. No other clinical signs, such as pruritus or cutaneous lesions, were observed. The macroscopic alterations in the affected remiges were characterized by the following appearance of the feather’s calamus: reduced diameter, length, and shape, along with complete absence of the superior umbilicus. Some rachises (shafts) exhibited persistent necrotic vascular structures, which were strongly adhered to the shaft and were associated with excessive keratin deposition and retention of the feather sheath (Figure 1). Similar alterations were observed in the tail feathers, with additional follicular dilation (cystic appearance) containing amorphous keratinous material and necrotic tissue (Figure 1). 

### 3.2. Serological Examination

The serum samples from the two animals included in the study tested negative for all the viral agents that were evaluated.

### 3.3. Microscopical Examination 

**Figure 2:**
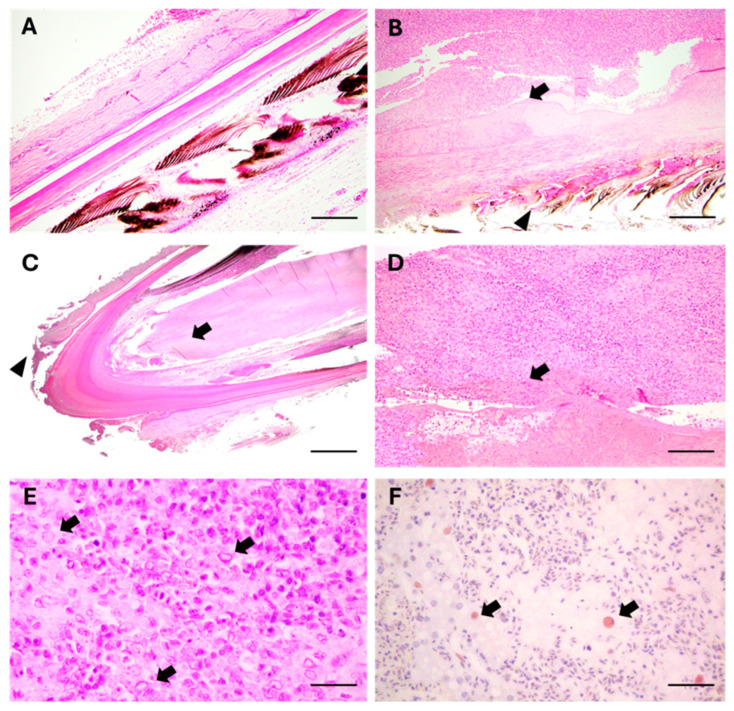
Histological aspects of dystrophic feathers from a griffon vulture (*Gyps fulvus fulvus*). Comparison of (**A**) a normal feather and (**B**) a dystrophic feather sampled at the same level and in the same anatomical area, from a healthy and a deplumed vulture. In the diseased bird, necrosis in the calamus pulp (arrows) and dystrophy of barbs and barbules (arrowheads) are evident (H&E stain, scale bar = 15 μm). (**C**) Complete view of the calamus of a dystrophic feather showing the dystrophic–necrotic aspect of the pulp area of the calamus (arrows) and the presence of dermatitis in the residual, perifollicular tissue (arrowheads) (H&E stain; scale bars = 1 mm). (**D**) Histology of the rachis, at the level of the calamus, of the dystrophic feather. High magnification of a pathological area of the calamus pulp, with associated inflammation (lymphohistiocytic infiltration) (circle) surrounding the necrotic–eosinophilic material (arrows) (H&E stain; scale bars = 0.5 mm). (**E**) Numerous cells in the calamus pulp show intra-nuclear inclusion bodies (arrows) (H&E stain; scale bars = 150 µm). (**F**) Several inclusions show weak to moderate immunolabeling to APV-VP-1 (arrows) (IHC stain, Harris’s hematoxylin nuclear counterstain; scale bars = 150 µm).

A histological comparison was conducted between dystrophic feathers from an affected vulture and normal feathers from a healthy animal (dead due to trauma), sampled at the same level and in the same area. In diseased birds, perifollicular, multifocal, chronic lympho-histiocytic dermatitis was observed, associated with fibrinoid necrosis, microvascular occlusion-thrombosis, and some nuclear inclusions, which were also reported in the endothelial cells (Figure 2A,B). Histological examination of the affected feathers revealed a condition of feather dystrophy primarily characterized by severe cell death in the pulp at the calamus level (Figure 2C), along with involvement of the feather’s branches and proximal barbules, at the level of the barbs (Figure 2D). The principal histological features of the dystrophic changes included the multifocal necrosis of pulp cells containing amphophilic intranuclear inclusions (Figure 2E); diffuse cell death at the base of the feather follicle, in the epidermal cells surrounding the dermal papilla and forming the collar; and severe lympho-histiocytic peri-necrotic pulpitis. Additional histopathologic changes included lympho-histiocytic vasculitis of variable severity, ranging from simple swelling and intracellular edema, involving the endothelial cells, to vasculitis with fibrinoid necrosis and micro-thrombotic events. Affected capillaries were located immediately adjacent to and within the level of the follicular dermal papilla, near the basal layer of the feather follicle. Both superficial and deep perifollicular lymphocytic inflammation were observed, with the accentuation of lesions at the calamus interface. The inflammatory infiltrates in peri-necrotic areas consisted mainly of lymphocytes and histiocytes. Cells interspersed within necrotic regions of the feather pulp, as well as scattered endothelial cells of damaged peri-follicular capillaries, displayed characteristic large amphophilic intranuclear inclusion bodies. In summary, the condition can be described as severe, diffuse, chronic lympho-histiocytic folliculitis and vasculitis accompanied by pulp necrosis.

### 3.4. Immunohistochemical Examination 

Immunohistochemical analysis using an anti-avian polyomavirus (APV) viral protein (VP)-1 antibody [20] revealed positive immunolabelling in several inclusion bodies (Figure 2F). The same histological sections, marked using the anti-avian *herpesvirus* ILTV antibody, gave consistently negative results. 

### 3.5. Ultrastructural Examination

**Figure 3:**
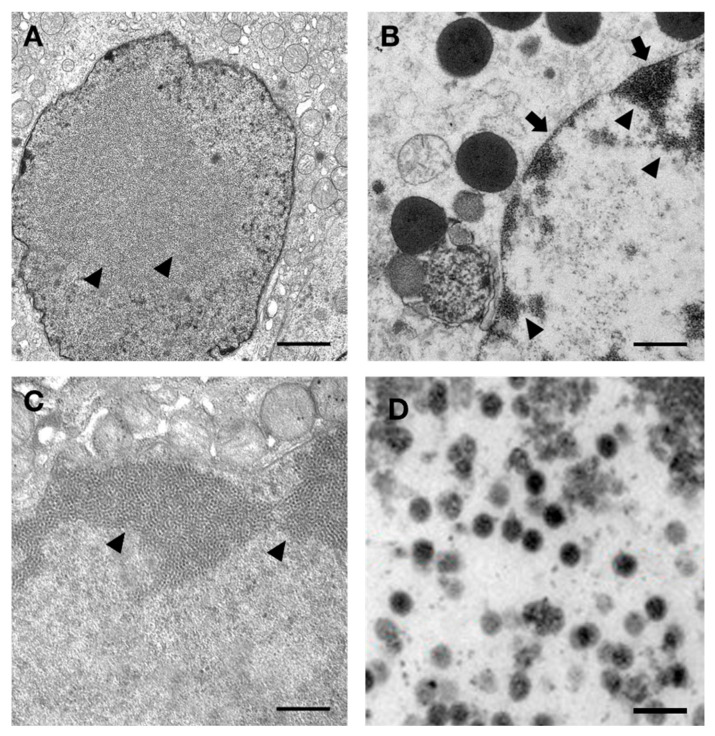
Electron micrographs of one of the altered and immunohistochemically VP-1 positive cell nuclei, observed in the area of necrotic calamus pulp. (**A**) The nucleus is increased in size with marginated or fragmented chromatin, with small paracrystalline structures aggregated in the nucleoplasm (arrowheads) (scale bar = 1.25 µm). (**B**) Perinuclear chromatin margination (arrow), homogeneous cytoplasmic appearance with viral-like particles lining the plasma membrane (arrowhead), and early degradation of mitochondria showing initial crystolysis (scale bar = 1.25 µm). (**C**,**D**) Higher magnification of paracrystalline structures aggregated in the nucleoplasm and composed of regular, symmetrical, icosahedral viral-like particles, (arrowheads) (scale bars: 0.25 µm (**C**); 0.05 µm (**D**)).

Transmission electron microscopy (TEM) was performed at the level of the calamus pulp, where necrosis and structural changes were mostly present. TEM revealed the presence of enlarged nuclei with marginated or fragmented chromatin and small paracrystalline aggregates in the nucleoplasm (Figure 3A). The paracrystalline aggregates were composed of regular, symmetrical, icosahedral particles morphologically compatible with virus-like elements. (Figure 3B,C). Cytopathic effects were characterized by perinuclear chromatin margination, a homogeneous cytoplasmic appearance with viral-like particles lining the plasma membrane, and early degradation of organelles, particularly mitochondria. Intranuclear inclusions were also observed within the endothelial cells.

### 3.6. Virome Analysis 

**Figure 4:**
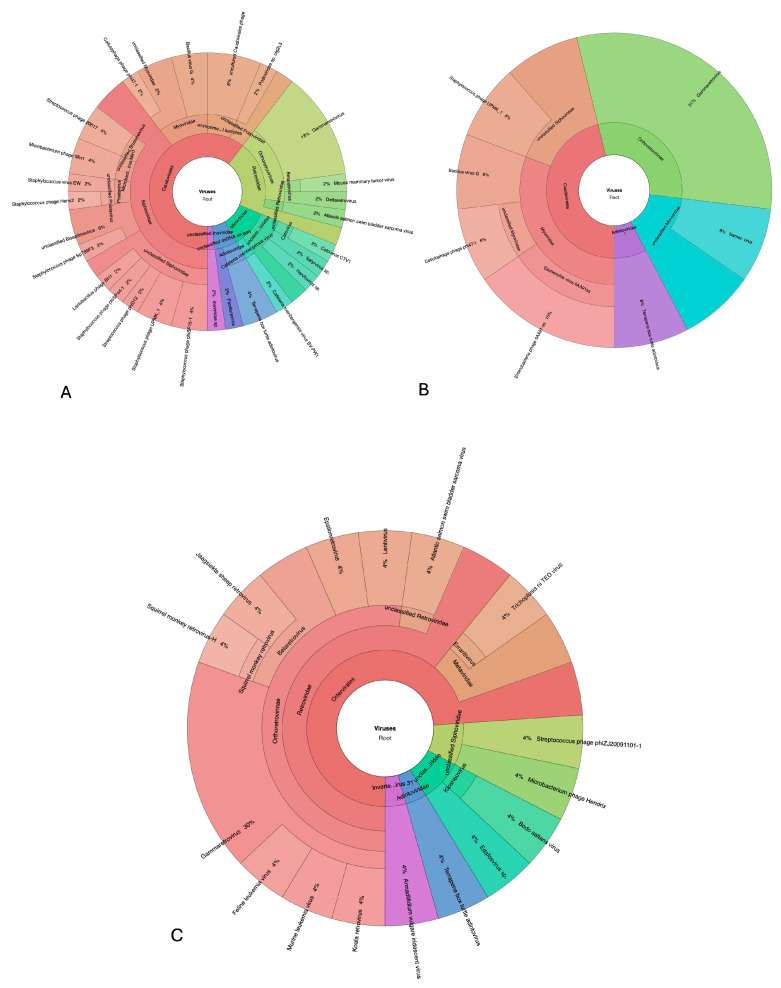
Taxonomy distribution of sequencing contigs obtained from pooled samples: feathers (**A**), blood (**B**), and calamus (**C**).

A total of 16,180,120 raw read sequences were obtained from sequencing samples. After the first cleaning step, a total of 12,734,568 were subjected to virome analysis. A small percentage of contigs (nr) were assigned to viruses against the NCBI nonredundant protein (nr) database using DIAMOND software v2.0.13 (26% for feather, 41% for calamus, and 33% for blood pooled samples) (Figure 4). A major part of viruses was classified as bacteriophages in the *Caudovirales* order in both blood and feather samples. However, a great part of contigs were attributed to the *Retroviridae* family in calamus (61% of viruses) compared to feather (21%) and blood (31%) pooled samples. No contigs aligning to virus reference genomes belonging to the *Polyomaviridae* family (Taxonomy ID: 151341) were obtained.

## 4. Discussion

Few cases of feather loss and abnormalities have been previously reported in free-living birds of prey [9,10,11,26]. These pathological conditions are typically associated with Pinching Off Syndrome (POS), which is characterized by the loss of one or more malformed primaries or rectrices feathers [26,27]. The etiology of this pathological condition remains unclear [9], though it has been associated with viral infections such as Psittacine Beak and Feather Disease (PBFD) or West Nile Virus (WNV) [10,11]. The macroscopic findings observed in two griffon vultures (*Gyps fulvus fulvus*) represent the first documented cases of POS in free-living Old and New World vultures. In contrast to previous unpublished reports describing feather abnormalities in pre-fledgling birds still confined to the nest, the clinical manifestations in the affected vultures were not limited to young or fledgling birds [9]. Another notable macroscopic deviation from the data reported by Muller et al. [9] is the absence of involvement of contour feathers in the pathological process. Feather loss, dystrophic growth, and cystic follicular formations were exclusively observed in the remiges and rectrices, and, as reported by other authors, feather quality continued to deteriorate after molting [9]. As previously mentioned, the observed lesions involved the dermal papilla, a critical functional area of the follicle, as the collar cells typically proliferate there to generate keratinocytes, including those in the ramogenic zone, which eventually differentiate into the barb and rachidial ridges [28]. All newly developing primary feathers and rectrices exhibited significant abnormalities, thus preventing the release of birds into the wild. Such feathering problems are a major concern to researchers involved in conservation and reintroduction programs for large birds of prey in their natural habitats, as the altered plumage can jeopardize the survival of the individuals born or released in the wild. Populations of large birds of prey, apical animals in the food chain, are facing a serious threat to their survival across the globe, attributable to numerous factors associated with human activity. Reintroduction strategies for endangered species, such as the griffon vulture, are pivotal in ensuring the preservation of ecological equilibrium. These birds play a vital role as scavengers, helping to control the populations of dead animals, preventing the spread of diseases, and maintaining a healthy, balanced ecosystem that promotes biodiversity and supports other species. Their ability to survive and reproduce strictly depends on the presence of optimal physical fitness and plumage. In the two cases of POS described in this study, histological analysis revealed a lesion pattern that overlaps with the one typically observed in parrots affected by avian polyomavirus (APV). This includes marked necrosis of the epithelial cells lining the feather follicles, accompanied by a pronounced inflammatory response characterized by immune cell infiltration into the affected areas and large basophilic-amphophilic intranuclear inclusion bodies within the epithelial cells in the necrotic pulp [2,29]. APV, along with other viruses such as *Gallid herpesvirus* (agent of Mareks’ disease) and *Circovirus*, are frequently associated with feather dystrophy in birds. These viruses typically initiate lesions at the vascular pole of the feather follicle and, in many cases, secondarily affect the stem cell population within the follicle [30,31]. Indeed, many viruses rely on undifferentiated or stem cell substrates with high proliferative activity for effective replication. As a result, the lesion is characterized as dystrophic, resulting in abnormal feather development due to significant disruption of the stem cell compartment, which often results in the improper eruption of feathers, leading to the formation of keratin cysts, nodules, or accumulations of keratin and feather sheath material. These changes can trigger a secondary inflammatory response, most commonly of a pyogranulomatous nature, although heterophilic inflammation can also occur. This inflammation resembles a type IV hypersensitivity reaction and is marked by intense macrophage activation. In the examined griffon vultures, the inflammatory patterns observed in the perivascular or intramural areas of the feather follicles, as well as in the medullary region of the feather shaft, reflect a post-necrotic and post-dystrophic reactive process. These findings support a similar viral pathogenesis. Specifically, APV DNA has been previously detected in raptors and extracted only from common kestrels (*Falco tinnunculus*) and common buzzards (*Buteo buteo*) without associated pathological feather abnormalities [18,32]. However, in this study, virome analysis did not confirm the presence of this virus in pooled affected samples, probably due to the inability to obtain fresh frozen samples from which viral DNA could be extracted. Nonetheless, the involvement of the APV in the lesions observed in the two studied vultures is further supported by the detection of several intranuclear inclusions that tested positive using a primary anti-VP-1 antibody [20]. This correlation is further reinforced by the presence of large clusters of viral-like particles within the nuclei, as observed through electron microscopy. Monitoring of the colony is ongoing, with efforts focused on amplifying the viral genome to confirm the presence and APV involvement in the disease.

## 5. Conclusions

This study reports the first cases of feather dystrophy in two free-living griffon vultures. Histological and ultrastructural findings suggest the involvement of a polyomavirus-like agent, although sequencing did not confirm this. Further studies are needed in order to confirm the hypothesis of the polyoma-like virus and to clarify the pathogen involved in the etiopathogenesis of this emerging plumage disorder in free-ranging griffon vultures.

## Data Availability

Sequencing data produced in this study have been deposited in Genbank SRA NCBI Database under BioProject accession number PRJNA1256769.

## Abbreviations

The following abbreviations are used in this manuscript:

POS	Pinching Off Syndrome
APV	Avian polyomavirus
VP	Viral protein
TEM	Transmission electron microscopy
PBFD	Psittacine Beak and Feather Disease
WNV	West Nile Virus
ILTV	Laryngotracheitis alpha1-*herpesvirus*
RT	Room temperature
HE	Hematoxylin and Eosin

## References

[1] Foth C., Foth C., Rauhut O.W.M. (2020). Introduction to the Morphology, Development, and Ecology of Feathers. The Evolution of Feathers.

[2] Ritchie B.W., Harrison L.R. (1994). Avian Medicine: Principles and Application.

[3] Oglesbee B.L. (1992). Hypothyroidism in a Scarlet Macaw. J. Am. Vet. Med. Assoc..

[4] Greenacre C.B., Latimer K.S., Niagro F.D., Campagnoli R.P., Pesti D., Ritchie B.W. (1992). Psittacine Beak and Feather Disease in a Scarlet Macaw (*Ara macao*). J. Assoc. Avian Vet..

[5] Perry R.A. (1981). A Psittacine Combined Beak and Feather Disease Syndrome with Particular Reference to the Australian Cockatoos *Cacatua galerita* (Sulphur-Crested Cockatoo), *Cacatua leadbeateri* (Major Mitchell or Pink Cockatoo), *Cacatua roseicapella* (Galah or Rose-Breasted Cockatoo) and *Cacatua Sanguinea* (Little Corella). Proceedings.

[6] McOrist S., Black D.G., Pass D.A., Scott P.C., Marshall J. (1984). Beak and Feather Dystrophy in Wild Sulphur-Crested Cockatoos (*Cacatua galerita*). J. Wildl. Dis..

[7] Melville D.S., Schuckard R. (2021). New Zealand King Shag (*Leucocarbo carunculatus*) with Deformed Primary Feathers. Notornis.

[8] Pass D.A., Plant S.L., Sexton N. (1994). Natural Infection of Wild Doves (*Streptopelia senegalensis*) with the Virus of Psittacine Beak and Feather Disease. Aust. Vet. J..

[9] Müller K., Schettler E., Gerlach H., Brunnberg L., Hafez H.M., Hattermann K., Johne R., Kollmann R., Krone O., Lierz M. (2007). Investigations on the Aetiology of Pinching off Syndrome in Four White-Tailed Sea Eagles (*Haliaeetus albicilla*) from Germany. Avian Pathol..

[10] Nemeth N.M., Kratz G.E., Bates R., Scherpelz J.A., Bowen R.A., Komar N. (2009). Clinical Evaluation and Outcomes of Naturally Acquired West Nile Virus Infection in Raptors. J. Zoo Wildl. Med..

[11] Sarker S., Lloyd C., Forwood J., Raidal S.R. (2015). Forensic Genetic Evidence of Beak and Feather Disease Virus Infection in a Powerful Owl, *Ninox Strenua*. Emu.

[12] Destro G.F.G., De Marco P., Terribile L.C. (2018). Threats for Bird Population Restoration: A Systematic Review. Perspect. Ecol. Conserv..

[13] Willette M., Rosenhagen N., Buhl G., Innis C., Boehm J. (2023). Interrupted Lives: Welfare Considerations in Wildlife Rehabilitation. Animals.

[14] Wink M. (1995). Phylogeny of Old and New World Vultures (Aves: *Accipitridae* and *Cathartidae*) Inferred from Nucleotide Sequences of the Mitochondrial Cytochrome b Gene. Z. Naturforschung C.

[15] Griffon Vulture Gyps fulvus Species. https://datazone.birdlife.org/species/factsheet/griffon-vulture-gyps-fulvus.

[16] Dobrev D., Tsiakiris R., Skartsi T., Dobrev V., Arkumarev V., Stara K., Stamenov A., Probonas N., Kominos T., Galanaki A. (2022). Long-Term Size and Range Changes of the Griffon Vulture *Gyps fulvus* Population in the Balkans: A Review. Bird Conserv. Int..

[17] Khan U., Murn C. (2011). Gyps Vulture Restoration Project—Role of Captive Breeding in Endangered Species Management. J. Anim. Plant Sci..

[18] Lorenzo-Betancor O., Galosi L., Bonfili L., Eleuteri A.M., Cecarini V., Verin R., Dini F., Attili A.-R., Berardi S., Biagini L. (2022). Homozygous CADPS2 Mutations Cause Neurodegenerative Disease with Lewy Body-like Pathology in Parrots. Mov. Disord..

[19] Desantis S., Galosi L., Santamaria N., Roncarati A., Biagini L., Rossi G. (2021). Modulation of Morphology and Glycan Composition of Mucins in Farmed Guinea Fowl (*Numida meleagris*) Intestine by the Multi-Strain Probiotic Slab51^®^. Animals.

[20] Rossi G., Taccini E., Tarantino C. (2005). Outbreak of Avian Polyomavirus Infection with High Mortality in Recently Captured Crimson’s Seedcrackers (*Pyrenestes sanguineus*). J. Wildl. Dis..

[21] Langmead B., Salzberg S.L. (2012). Fast Gapped-Read Alignment with Bowtie 2. Nat. Methods.

[22] Li H., Handsaker B., Wysoker A., Fennell T., Ruan J., Homer N., Marth G., Abecasis G., Durbin R., 1000 Genome Project Data Processing Subgroup (2009). The Sequence Alignment/Map Format and SAMtools. Bioinformatics.

[23] Bankevich A., Nurk S., Antipov D., Gurevich A.A., Dvorkin M., Kulikov A.S., Lesin V.M., Nikolenko S.I., Pham S., Prjibelski A.D. (2012). SPAdes: A New Genome Assembly Algorithm and Its Applications to Single-Cell Sequencing. J. Comput. Biol..

[24] Buchfink B., Xie C., Huson D.H. (2015). Fast and Sensitive Protein Alignment Using DIAMOND. Nat. Methods.

[25] Ondov B.D., Bergman N.H., Phillippy A.M. (2011). Interactive Metagenomic Visualization in a Web Browser. BMC Bioinform..

[26] Cooper J.E. (1985). Veterinary Aspects of Captive Birds of Prey.

[27] Cooper J.E. (1972). Feather Conditions in Birds of Prey. J. N. Am. Falconers’ Assoc..

[28] Yu M., Yue Z., Wu P., Wu D.-Y., Mayer J.-A., Medina M., Widelitz R.B., Jiang T.-X., Chuong C.-M. (2004). The Developmental Biology of Feather Follicles. Int. J. Dev. Biol..

[29] Phalen D.N. (2008). Papillomaviruses and Polyomaviruses. Infectious Diseases of Wild Birds.

[30] Couteaudier M., Trapp-Fragnet L., Auger N., Courvoisier K., Pain B., Denesvre C., Vautherot J.-F. (2015). Derivation of Keratinocytes from Chicken Embryonic Stem Cells: Establishment and Characterization of Differentiated Proliferative Cell Populations. Stem Cell Res..

[31] Couteaudier M., Courvoisier K., Trapp-Fragnet L., Denesvre C., Vautherot J.-F. (2016). Keratinocytes Derived from Chicken Embryonic Stem Cells Support Marek’s Disease Virus Infection: A Highly Differentiated Cell Model to Study Viral Replication and Morphogenesis. Virol. J..

[32] Johne R., Müller H. (1998). Avian Polyomavirus in Wild Birds: Genome Analysis of Isolates from Falconiformes and Psittaciformes. Arch. Virol..

